# Possible giant cell arteritis symptoms are common in newly diagnosed patients with Polymyalgia Rheumatica: results from an incident primary care PMR cohort

**DOI:** 10.1186/s41927-017-0007-2

**Published:** 2017-12-13

**Authors:** William Masson, Sara Muller, Rebecca Whittle, James Prior, Toby Helliwell, Christian Mallen, Samantha L. Hider

**Affiliations:** 1 Arthritis Research UK Primary Care Centre, Primary Care Sciences, Keele University, Keele, Staffordshire, ST5 5BG UK; 2 Rheumatology Department, Haywood Rheumatology Centre, Staffordshire, ST6 7AG UK

**Keywords:** Polymyalgia rheumatica, Giant cell arteritis, Headache

## Abstract

**Background:**

To examine the frequency of possible giant cell arteritis (GCA) symptoms (including headache, temporal/scalp tenderness, jaw claudication and visual symptoms) in newly diagnosed polymyalgia rheumatica (PMR) patients in UK primary care.

**Methods:**

The PMR Cohort Study is a primary care inception cohort of 652 adults with newly diagnosed polymyalgia rheumatica (PMR). At baseline, participants were asked to report (yes/no) on the presence of seven potential GCA symptoms: sudden headache, tender scalp, disturbed/double vision, jaw claudication, fever, appetite loss and unintentional weight loss.

**Results:**

Of the 652 patients, 405 (62%) were female, with a mean (SD) age of 72.5 (8.9) years. Sudden headache was the commonest symptom in 161 patients (24.7%). The least commonly reported symptom was jaw claudication in 66 (10.1%) patients. Females had a higher prevalence of headache, tender scalp and jaw pain. Sudden onset headache and fever were commoner in younger patients, (OR (95% CI) per 10 year age band increase: headache 0.76 (0.62–0.92), fever 0.63 (0.49, 0.79)). In those reporting sudden headache (*n* = 161), 19.9% (*n* = 32) also reported double/disturbed vision and a tender scalp, whilst 11.8% (*n* = 19) reported double/disturbed vision and jaw pain.

**Conclusion:**

The data suggests possible GCA symptoms are common in PMR patients, particularly sudden headache, appetite loss and weight loss. These symptomatic PMR patients warrant careful monitoring and consideration for early referral to specialist services.

**Electronic supplementary material:**

The online version of this article (10.1186/s41927-017-0007-2) contains supplementary material, which is available to authorized users.

## Background

Giant cell arteritis (GCA, or temporal arteritis) is a systemic large vessel vasculitis with a tendency to affect the aortic and extracranial branches, such as superficial temporal arteries [1]. GCA affects older adults (typically those >50 years) with an estimated annual incidence of 1 per 10,000 per year in those over 40 years [2]. Its aetiology is currently unknown, other than it is an immune mediated disease which has a clear association with polymyalgia rheumatica (PMR) and females are affected up to three times more frequently than males [3].

Prompt diagnosis and treatment of GCA is essential to prevent complications such as irreversible blindness [4]. Common symptoms of GCA include headache, temporal/scalp tenderness, jaw claudication and diplopia. However, studies suggest that there may be an over-reliance on headache as a presenting feature of GCA with one study suggesting a prevalence of headache of 72%, [5], but with audit data suggesting those without headache are at increased risk of visual loss, perhaps as a consequence of delayed recognition [6]. It is especially important for those with PMR to be monitored for signs and symptoms of GCA, as it has been reported in secondary care populations that between 16 and 21% [3, 7, 8] of those with PMR will develop GCA at some point in their disease course. However, this may be different in community populations with PMR, and it is not clear to what extent this overlap is present in the community setting. This study aims to describe the prevalence of common symptoms that are potentially indicative of GCA in a cohort of English adults with incident PMR diagnosed in primary care.

## Methods

### Study design

The PMR Cohort Study is a cohort of 652 adults with newly diagnosed PMR recruited in primary care. The study has been described in detail elsewhere [9, 10]. Briefly, GP’s entering a first Read Code for PMR were invited to refer patients into the study and encouraged to request the recommended blood tests. All participating general practices were provided with copies of the British Society for Rheumatology guidelines for the management of PMR [11]. Between June 2012 and June 2014, patients with a new primary care diagnosis of PMR were referred into the study by their GP and mailed a baseline questionnaire. This included information regarding PMR symptoms (current pain and stiffness using a numeric rating scale (NRS)), lifestyle factors and socio-demographics. Participants were also asked to report (yes/no) on the presence of seven potential symptoms of GCA including sudden onset headache, tender scalp, disturbed/double vision, jaw pain on chewing, temperature/fever, appetite loss and unintentional weight loss. Following completion of the baseline questionnaire, participants were followed-up by postal questionnaire at regular intervals for two years, with medical record review at the end of the study in those consenting, for recorded symptoms, treatment and diagnoses. The current study uses data from the baseline questionnaire only.

### Statistical analysis

Descriptive statistics were used to describe the sample. The prevalence of GCA symptoms was calculated as a percentage. Comparisons of prevalence were made across genders and age (in 10-year age bands) using cross tabulation and odds ratios with 95% confidence intervals. The prevalence of pairs of GCA symptoms was also investigated using simple descriptive statistics. As headache is an important symptom of GCA, but is relatively common in the general non-PMR population, the prevalence of combinations of symptoms with headache were also investigated.

## Results

Of the 652 incident PMR cases the mean (SD) age was 72.5 (8.9) years and 405 (62%) were female. Table 1 shows the baseline demographics and clinical features of the cohort. The median (IQR) time from referral into the cohort to the baseline postal questionnaire being received was 16 (11, 23) days. Sudden onset headache (161, 24.7% patients), appetite loss (140, 21.5%) and unintended weight loss (137, 21%) were the most common symptoms (Table 2). Females were significantly more likely to report all symptoms than men, with the exception of unintentional weight loss, where there was no gender difference. (Table 2). Sudden onset headache and fever were the only symptoms significantly associated with age, with younger patients more likely to experience these symptoms (OR (95% CI) for headache per 10-year increase 0.76; 0.62–0.92) and fever (OR (95% CI) per 10-year increase 0.63 (0.49, 0.79)).

**Table 1:** Baseline Characteristics of the sample

		*N* = 652
Age (Mean, SD)		72.5 (8.9)
Gender (N %)		
Female		405 (62.1)
Male		247 (37.9)
Median (IQR) pain score (NRS) at presentation		8 (7, 9)
Median (IQR) stiffness score (NRS) at presentation		8 (7, 9)
Morning stiffness >45 min duration (n, %)		524 (80.3)
Current steroid dose for PMR treatment (mg/day)^a^ N (%)		
<10 mg		64 (12.3)
10 < 15 mg		87 (16.7)
15 < 20 mg		218 (41.9)
20 < 25 mg		111 (21.4)
25-30 mg		6 (1.2)
≥30 mg		34 (6.5)
Smoking (n, %)		
Never		317 (49.3%)
Previously		286 (44.5%)
Currently		40 (6.2%)

^a^ Data on current steroid dose not available for 132 patients. NRS- numerical rating scale, where 0 = none and 10 is very severe.

**Table 2:** Possible GCA Symptoms

	All (n = 652)	Female (*n* = 405)	Male (*n* = 247)	Odds Ratio (95% CI): females vs. males (ref. category)	Mean (sd) age with symptoms	Mean (sd) age no symptoms	Odds Ratio (95% CI): per 10 year age increase
Sudden headache	161 (24.7)	123 (30.4)	38 (15.4)	2.40 (1.60, 3.60)	70.8 (9.5)	73.1 (8.7)	0.76 (0.62, 0.92)
Appetite loss	140 (21.5)	102 (25.2)	38 (15.4)	1.85 (1.23, 2.80)	73.1 (9.2)	72.4 (8.9)	1.09 (0.88, 1.35)
Unintended weight loss	137 (21.0)	83 (20.5)	54 (21.9)	0.92 (0.63, 1.36)	73.3 (8.8)	72.3 (9.0)	1.14 (0.92, 1.42)
Tender scalp	123 (18.9)	87 (21.5)	36 (14.6)	1.60 (1.04, 2.45)	72.8 (8.9)	72.4 (9.0)	1.05 (0.84, 1.31)
Disturbed / double vision	110 (16.9)	84 (20.7)	26 (10.5)	2.22 (1.39, 3.57)	71.6 (9.5)	72.7 (8.8)	0.87 (0.70, 1.09)
Temperature/ fever	99 (15.2)	72 (17.8)	27 (10.9)	1.76 (1.10, 2.83)	69.2 (9.4)	73.1 (8.7)	0.63 (0.49, 0.79)
Jaw pain on chewing	66 (10.1)	50 (12.4)	16 (6.5)	2.03 (1.13, 3.66)	72.3 (8.0)	72.5 (9.0)	0.98 (0.74, 1.30)

All are N (%) unless otherwise stated

### Pairs of GCA symptoms

In terms of symptom pairs, headache and tender scalp were the most common combination pair of symptoms with a prevalence of 10.1%, followed by headache and double vision (9.5%) (Table 3). In those reporting sudden headache (*n* = 161) (Table 2), 19.9% (*n* = 32) also reported double/disturbed vision and a tender scalp, whilst 11.8% (*n* = 19) reported double/disturbed vision and jaw pain. Double/disturbed vision, tender scalp and jaw pain was reported by 13 individuals (8.1%) reporting sudden headache.

**Table 3:** Symptom combinations in those with PMR

Symptom Combinations	n (%)
Headache & Tender Scalp	66 (10.1%)
Headache & Double/Disturbed Vision	62 (9.5%)
Headache & Jaw Pain	39 (6.0%)
Headache & Tender Scalp & Jaw Pain	22 (3.4%)
Double/Disturbed Vision & Tender Scalp	41 (6.3%)
Double/Disturbed Vision & Jaw Pain	23 (3.5%)

## Discussion

This study of incident PMR cases suggests that classical GCA symptoms are common in newly diagnosed PMR patients, with 1 in 4 patients reporting sudden headache, appetite loss or unintentional weight loss. Jaw pain on chewing was the least common reported symptom. Except for unintentional weight loss, a higher proportion of females reported all symptoms, whilst sudden headache, double vision and fever were reported by fewer people at older ages. Given that older patients seem to be at greater risk of both developing GCA and of visual loss (7) it may be that reporting of these symptoms is more specific in older individuals, who may report fewer symptoms but be at greater risk of complications. The most common combination of symptoms was headache with tender scalp and headache with double vision, each affecting approximately 10% of the cohort at diagnosis. Further follow up of the cohort will determine the proportion of patients who were formally diagnosed as having GCA.

The PMR Cohort study is the first inception cohort study of PMR patients in primary care and is of a substantial size. Although primary care recruitment can be seen as a weakness, because no specialist opinion as to the diagnosis was sought, this is also a major strength of the study because the majority of patients with PMR are diagnosed and managed exclusively in primary care [12]. This sample is therefore free of the potential spectrum bias that is likely to be present in studies conducted in specialist settings where disease may be more severe, atypical or difficult to manage. Reassuringly however, the demographic and clinical characteristics of this cohort are similar to other secondary care cohorts [13], providing confidence in the accuracy of the primary care PMR diagnosis. Furthermore symptoms were recorded from patients close to the time of diagnosis thus reducing the recall bias.

One of the limitations of this study is that at this stage it is unknown if the patient was formally diagnosed with GCA or not. The prevalence of possible GCA symptoms was higher in women in our cohort, in common with the reported higher prevalence of GCA in women. [7]. Furthermore, the study demonstrates that in patients with PMR, headache is a much commoner symptom than in the older adult general population. Work by Steiner et al. [14] demonstrated that in the older adult general population (aged 50–65) the prevalence of headache was 3.4% for males and 13.5% for females The prevalence of headache was considerably higher in our cohort (males 15.4%, females 30.4%), suggesting that PMR is associated with an increase in reported headaches, which may reflect the overlap with giant cell arteritis, or that some cases of GCA are not being recognised or misdiagnosed. Although females have a higher prevalence of headaches overall, males saw a much bigger increase in the prevalence of headaches compared with a similar age general population group. However, a Danish population survey suggested the prevalence of headache to be 36.5%, although only 17% had consulted primary care because of headache symptoms [15]. Previous studies comparing patients with isolated PMR and those who went onto develop GCA have suggested that new onset headache is a key predictor [16, 17], with others suggesting that the headache is over-relied on in making a GCA diagnosis [5, 6].

Given that single symptoms such as headache are common both in the general population and in those with PMR it may be that combinations of symptoms are more useful in identifying patients at risk of GCA. Headache and tender scalp are two of the most common symptoms reported in those diagnosed with GCA and were reported by around 10% of this PMR Cohort. Further follow-up will assess whether these patients were initially misdiagnosed as PMR instead of GCA, together with further assessment of the utility of combinations of symptoms in predicting those patients at higher risk of GCA.

## Conclusions

In summary, within a cohort of primary care patients newly diagnosed with PMR, symptoms suggestive of possible GCA, such as headache, tender scalp and visual disturbance are common. These occur in more than 1 in 4 patients and at higher rates than in the older age UK general population. Given the risk of irreversible visual loss in those with untreated GCA it may be that PMR patients who have these symptoms warrant closer monitoring and follow up to ensure that GCA does not develop and that symptoms do not progress. Future follow-up of this cohort will enable greater understanding of the proportions of primary care PMR patients who develop GCA and the risk factors for GCA development. Enabling risk stratification of PMR patients could facilitate GP education (regarding patients at higher risk) and more effective referral or treatment interventions to reduce the impact of this potentially serious complication.

## Additional file

Additional file 1: Reviewer reports and AU response to reviewers. (DOCX 17 kb)

## Abbreviations

GCA: giant cell arteritis
GP: general practitioner
PMR: polymyalgia rheumatica
