# Supplementary material for: Possible giant cell arteritis symptoms are common in newly diagnosed patients with Polymyalgia Rheumatica: results from an incident primary care PMR cohort
Source: BMC Rheumatol. 2017 Dec 13;1:7. doi: 10.1186/s41927-017-0007-2 (PMC6390563; doi:10.1186/s41927-017-0007-2)
Supplement: Supplementary file 1 — Reviewer reports and AU response to reviewers. (DOCX 17 kb) [file 41927_2017_7_MOESM1_ESM.docx]

**Open Peer Review summary**

Pre-publication versions of this article, reviewer reports and author comments to reviewers are available by contacting [info@biomedcentral.com](mailto:info@biomedcentral.com).

| Original submission |  |  |
| --- | --- | --- |
| 24 May 2017 | Submitted | Original manuscript |
| 7 August 2017 | Reviewed | Reviewer report – Nader A. Khalidi |
| 21 September 2017 | Reviewed | Reviewer report – Sam Whittle |
| 25 October 2017 | Author responded | Author comments – Samantha L Hider |
| Resubmission – Version 2 |  |  |
| 25 October 2017 | Submitted | Manuscript version 2 |
| 29 October 2017 | Author responded | Author comments – Samantha L Hider |
| Resubmission – Version 3 |  |  |
| 29 October 2017 | Submitted | Manuscript version 3 |
| 9 November 2017 | Author responded | Author comments – Samantha L Hider |
| Resubmission – Version 4 |  |  |
| 9 November 2017 | Submitted | Manuscript version 4 |
| Publishing |  |  |
| 20 November 2017 | Editorially accepted |  |
| 13 December 2017 | Article published | 10.1186/s41927-017-0007-2 |

### How does Open Peer Review work?

Open peer review is a system where authors know who the reviewers are, and the reviewers know who the authors are. If the manuscript is accepted, the named reviewer reports are published alongside the article. Pre-publication versions of the article are available by contacting [info@biomedcentral.com](mailto:%20mailto:info@biomedcentral.com).

You can find further information about the peer review system at <https://bmcrheumatol.biomedcentral.com/about#peer-review+policy>
